# Transcriptomic Analysis of Gingival Tissues: Insights Into Gene Expression Profiles

**DOI:** 10.1155/ijog/1427141

**Published:** 2025-12-08

**Authors:** Saravanan Sampoornam Pape Reddy, Yash Dhiman, Delfin Lovelina Francis, Manish Rathi, Ankit Gupta, Ruchi Harish, Balaji Manohar, Shaili Pradhan

**Affiliations:** ^1^ Department of Periodontology, Army Dental Corps, New Delhi, India; ^2^ School of Biosciences, University of Birmingham, Edgbaston, UK, birmingham.ac.uk; ^3^ Department of Public Health Dentistry, Saveetha Dental College and Hospitals, Saveetha University, Saveetha Institute of Medical and Technical Sciences (SIMATS), Chennai, Tamil Nadu, India, saveetha.com; ^4^ Department of Periodontology, Army Dental Centre (Research & Referral), New Delhi, India; ^5^ Department of Periodontology, Geetanjali Dental and Research Institute, Geetanjali University, Udaipur, Rajasthan, India, geetanjaliuniversity.com; ^6^ Department of Periodontology, Kathmandu Medical College Public Limited, Kathmandu, Nepal

**Keywords:** computational biology, connective tissue, epithelium, gingiva, sequence analysis

## Abstract

**Objectives:**

The aim of this study was to understand the transcriptome and molecular pathways that drive unique functional characteristics of human gingiva.

**Materials and Methods:**

Gene expression data were obtained from the NCBI GEO database, including GSE38617 (connective tissue [CT]) and GSE7224 (gingival epithelium [GE]). Raw data were preprocessed and filtered for healthy samples. Differential gene expression analysis was performed using the limma package in R, with a significance cutoff of log_2_ fold change ≥ 1 and adjusted *p* value < 0.05.

**Results:**

The analysis highlighted 11,096 DEGs between the dissected tissues. In CT, 7564 genes were upregulated in extracellular matrix (ECM) organization, collagen synthesis, and immunomodulation. GE had a total of 3532 upregulated genes enriched in epithelial barrier integrity, antimicrobial defense, and keratinocyte differentiation. These patterns were confirmed using key markers (CT: COL1A1 and COL3A1 and GE: DEFB1 and KRT10). In CT, pathway enrichment showed PI3K–Akt signaling and ECM–receptor interaction, whereas GE was characterized by tight junctions and the IL‐17 signaling pathway.

**Conclusion:**

The differences in transcriptional landscapes of CT and GE illustrate specialized functions of each tissue type in maintaining periodontal health. Whereas CT focuses on ECM preservation and its immunomodulatory role, GE emphasizes antimicrobial defense and barrier function.

**Summary**

This study compared the transcriptomic profiles of healthy gingival epithelium and connective tissue and highlighted distinct gene expression patterns. Connective tissue upregulates extracellular matrix remodeling and structural support genes, whereas the gingival epithelium emphasizes barrier integrity and antimicrobial defense. Functional analyses revealed complementary roles, offering insights into periodontal health and potential therapeutic targets.

## 1. Introduction

The oral cavity comprises a number of highly specialized tissues involved in its homeostasis and protection against external challenges (i.e., microbial invasion, mechanical stress, and chemical stress) [1]. Of these tissues, the gingival epithelium (GE) is the first physical barrier and immunological barrier. It blocks harmful pathogens and environmental insults from entering the host, coordinating immune responses in the process via secretion of antimicrobial peptides (AMPs) and pro‐inflammatory mediators [2]. Connective tissue (CT) provides structural support, immune function, critical wound healing components, and repair functions beneath the GE. Interactions between GE and its underlying CT are critical in the creation and maintenance of periodontal homeostasis, as well as in response to insults. Despite their functional interdependence, the molecular features that distinguish GE from CT remain poorly understood. A molecular understanding of these differences will be critical for expanding our understanding of periodontal biology and pathobiology. It would be interesting to conduct comparative transcriptomic analyses of these tissues to identify additional differences in their gene expression profiles, which may point to their unique biological functions. Thus, such analyses may serve as a basis for discerning tissue‐specific pathways and regulatory networks that preserve periodontal health or alter disease pathogenesis when perturbed.

Emerging high‐throughput transcriptomic technologies (microarray analysis and RNA sequencing) have enabled the global profiling of gene expression in oral tissues. These technologies have proven valuable in elucidating pathways central to extracellular matrix (ECM) remodeling, immune regulation, epithelial barrier function, and antimicrobial defense. Prior studies have indicated that CT is enriched for genes related to collagen biosynthesis, ECM organization, and immune modulation, whereas GE is characterized by the expression of genes governing epithelial differentiation, keratinization, and antimicrobial activity. These results highlight the tissue‐specific roles of each line in preserving periodontal homeostasis and responding to external stimuli [3].

Periodontitis, a chronic inflammatory disease, highlights the need to understand these tissue‐specific molecular profiles. This condition is induced by dysbiotic oral microbiota that elicits a hyperbolic host immune response resulting in progressive destruction of the gingival and CTs [4]. The disease is linked to major transcriptomic reprogramming of both tissues, with marked involvement of immune signaling, ECM remodeling, and epithelial barrier penetration. However, detailed baseline molecular profiles of healthy GE and CT are critical for placing these shifts in disease context and for discerning potential therapeutic targets [5].

In this study, we compared the transcriptomic landscapes of healthy GE and CT using publicly available datasets. Through differential expression of genes (DEGs), pathway enrichment analyses (PEA), and gene ontology (GO) term classification, we aimed to determine the biological processes and molecular pathways that characterize the distinct functions of these tissues. The data from this study enhance our understanding of the molecular pathways involved in the homeostasis of the periodontium and provide a foundation for future exploration of pathophysiological processes during periodontitis and therapeutic approaches.

## 2. Material and Methods

### 2.1. Data Acquisition and Preprocessing

Gene expression data for healthy GE and CT were downloaded from the NCBI Gene Expression Omnibus (GEO) database. The selected datasets were GSE38617 (CT) and GSE7224 (GE). Healthy tissue samples were selected from each dataset based on the metadata provided by individual studies. The GEOquery package was used to download raw expression data in R, which were log‐transformed to normalize the data and remove variability. Batch effects due to different studies running under nonidentical conditions using different sequencing platforms or data‐processing pipelines were corrected using the “ComBat” algorithm provided in the “sva” package. This ensured that differences in observed gene expression profiles were representative of biological heterogeneity, as opposed to technical artifacts.

We also performed a sensitivity analysis to characterize whether our significant findings were robust and not an artifact of the batch correction used. In addition to the primary analysis using the remove “Batch Effect” of the limma package, we also employed an alternative method that has been widely used together with ComBat batch correction from the sva package. The combined, un‐normalized expression matrix was batch corrected using ComBat with a model matrix that included the tissue of interest (“tissue”) as a biological variable and the “batch” variable, which represented the study of origin (GSE38617/GSE7224). After this modification, the differential expression analysis was run again with the limma pipeline. We compared the highest 200 DEGs in both limma and ComBat‐corrected datasets demonstrating a significant overlap, which validated our results and ensured that these are independent of the batch correction methodology used. The GSE38617 dataset was obtained from biopsies of gingival CT from eight healthy donors (mean age: 36 ± 5 years) using the Affymetrix Human Genome U133 Plus 2.0 Array platform. We prepared the GSE7224 dataset from six human donor‐derived GE biopsies, which was generated via the Agilent‐014850 Whole Human Genome Microarray platform (mean age: 34 ± 4 years). Sample collection methods, RNA extraction procedures, and array platforms were also different across the original studies; these differences might introduce batch effects or other sources of nonbiological variability. To eliminate these differences, normalization and batch effect correction were performed. First, raw CEL and TXT files were imported into R using the GEOquery package, background‐corrected, and log_2_‐transformed. We mapped probe identifiers to gene symbols using annotations specified by the appropriate platform specification and only kept genes that were common in both datasets. The method we have used to account for batch effects was the empirical Bayes approach, and it was implemented in ComBat (sva package). Unsupervised analyses of PCA before correction revealed clustering based clearly on platform, which disappeared in postcorrection analysis, thus confirming the effectiveness of batch effect removal. To test the robustness of our findings, we also used the remove unwanted variation (RUV) method as an alternative batch effect correction method. The DEG and pathway enrichment conclusions from RUV‐corrected data were in tight concordance with ComBat‐corrected results (Pearson correlation of log_2_ fold‐change values = 0.93), providing evidence that any remaining technical noise did not appreciably affect focal analyses.

### 2.2. Gene Selection

This refocuses on the analysis of biologically relevant genes based on key markers corresponding to the different functions of CT and GE. For CT, COL1A1 and COL3A1 were selected because of their known functions in ECM remodeling and structural support. However, DEFB1 and KRT10 were chosen based on their roles in antimicrobial defense, barrier integrity, and keratinocyte differentiation relevant to GE. Therefore, these genes serve as initial biological anchors that could help to understand the molecular differences between the two classes of tissues.

### 2.3. Data Integration and Merging

A combination of the normalized CT and GE datasets into a single dataset was performed after preprocessing. This also included mapping each dataset′s probe identifiers to a common gene identifier (e.g., gene symbols). Genes common to both datasets were retained for the analysis. By integrating the data in this fashion, a direct comparison between healthy CT and GE transcriptomic profiles could be conducted, and a broader perspective of gene expression across the two tissue types could be gained.

### 2.4. Differential Gene Expression Analysis

DEG analysis between CT and GE was performed using a two‐sample *t*‐test approach to identify DEGs. More specifically, the R package “limma” was used to compare the mean expression levels between groups for every gene in the merged dataset. Genes were classified as “differentially expressed” if they displayed a fold change greater than 2 or a fold change lower than −2 and an adjusted *p* value lower than 0.05. Thus, the use of this dual criterion guaranteed that the chosen DEGs were both biologically relevant (i.e., had a significant fold change) and statistically robust (i.e., satisfied the adjusted significance threshold).

### 2.5. Pathway Enrichment and Functional Analysis

**Figure 1:**
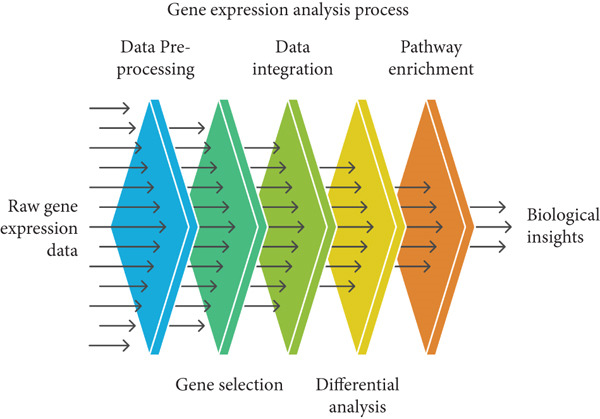
Steps utilized in bioinformatic methodology.

The biological functions of the identified DEGs were explored by performing PEA [6]. The DEGs were mapped to the related biological processes, molecular functions, and KEGG pathways using the “clusterProfiler” package in R, followed by enrichment analysis according to Fisher′s exact test to determine the overrepresentation of DEGs in a certain pathway. Benjamini–Hochberg‐adjusted *p* values were computed to correct for multiple comparisons [7]. The significantly enriched pathways that differ in both types of tissue provide insight into the biological distinction between CT and GE (Figure 1).

## 3. Results

Different techniques were used to visualize the findings from the analysis as they efficiently conveyed the outcome. Volcano plots were constructed to visualize the magnitude and significance of DEGs, with genes that had the largest fold changes and smallest *p* values in the foreground. The enriched pathways were plotted as bar charts and bubble charts, with bar charts representing overrepresented biological processes across each tissue type. Heatmaps provided insight into the gene expression profiles, especially for those with the highest differential expression, which could be plotted together to provide a rapid visual reference for relatively higher or lower expression in CT and GE. To accomplish this goal, the visualization methods presented here illustrate the major results from the analysis, including the genes and pathways uniquely upregulated within CT as compared with GE to serve the purpose of this investigation in uncovering the molecular foundations of normal periodontal tissues.

### 3.1. Statistical Tools and Software

Data processing, analysis, and visualization were performed using R, a widely used statistical programming environment. Key packages were “sva,” which reduces technical variability between studies for batch effect correction using “ComBat.” Differential expression analysis was performed using “limma,” employing its two‐sample comparison method. For pathway enrichment and GO analysis, “clusterProfiler” was used as defined previously [8, 9]. Customizable, high‐quality plots to present the study findings were developed in R using “ggplot2” as described previously [10]. Through an integrated method framework that included (1) data acquisition and preprocessing, (2) batch correction, (3) gene selection, (4) differential expression, and (5) pathway enrichment, a robust and systematic analysis of gene expression profiles of healthy CT and GE, and, hence, their molecular and functional differences, was addressed as per the previously described methodology [11].

### 3.2. Data Overview and Quality Assessment

Healthy tissue samples were identified and filtered from GSE38617 (CT) and GSE7224 (GE). Both datasets were log_2_‐transformed and adjusted for batch effects using ComBat, which resulted in a quasiperfect distribution profile. These checks provided additional confidence that no significant technical artifacts would be present for downstream comparisons.

### 3.3. Differential Gene Expression Analysis

After aligning gene identifiers and merging the two datasets, we conducted a two‐sample *t*‐test (using the limma package) to calculate DEGs between healthy CT and GE. A log_2_ fold change ≥ 1 (≥ 2‐fold) and adjusted *p* value < 0.05 were considered significant.

### 3.4. Overall DEGs

**Figure 2:**
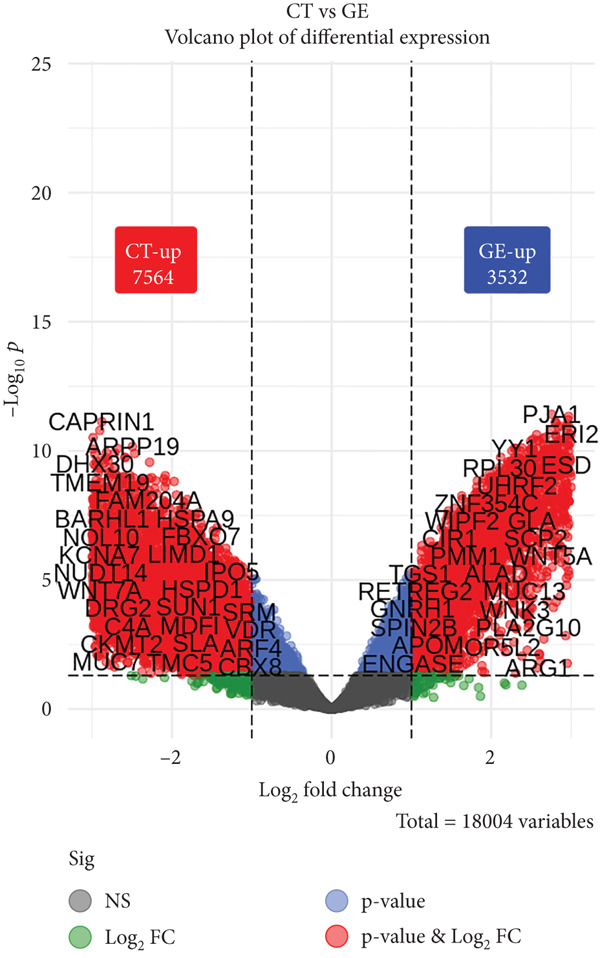
Volcano plot showing overall differentially expressed genes.

A total of 11,096 out of 14,084 shared genes were identified as DEGs. There were 7564 genes with higher expression in the CT (negative logFC in the GE–CT contrast). A total of 3532 genes were upregulated in the GE (positive logFC). The volcano plot (Figure 2) was used to show the log_2_ fold change (*x*‐axis) against −log_10_(*p* value) (Fisher scale on *y*‐axis). The left point frame shows a greater concentration of points in the CT range, indicating that CT has a more advanced upregulated gene pattern. Genes on the left represent those highly expressed in GE. Asymmetry is underscored with numeric annotations (7564 for CT‐up and 3532 for GE‐up).

### 3.5. Heatmap of Top 50 DEGs and Marker Genes

**Figure 3:**
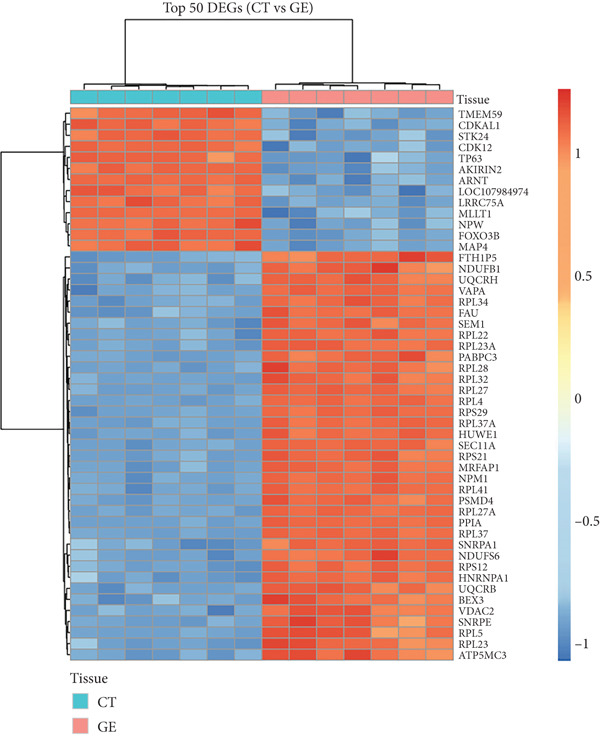
Heatmap of Top 50 differentially expressed genes.

A heatmap of the 50 DEGs that were most abundant (Figure 3) provided deeper insight into patterns of gene expression: CT samples were defined by upregulation of ECM and collagen‐related genes (consistent with the role of both COL1A1 and COL3A1 as structural proteins). Compared with the rest of the oral epithelium, GE had upregulated expression of antimicrobial and keratinocyte differentiation genes (DEFB1 and KRT10), mirroring the defense and barrier functions of the tissues, indicating that distinct transcriptomic scenery exists for each of these tissues.

### 3.6. Pathway Enrichment and Functional Insights

Subsequently, the clusterProfiler package was used for GO and KEGG PEA to elucidate the functional implications of these DEGs.

### 3.7. GO Biological Process Enrichment

**Figure 4:**
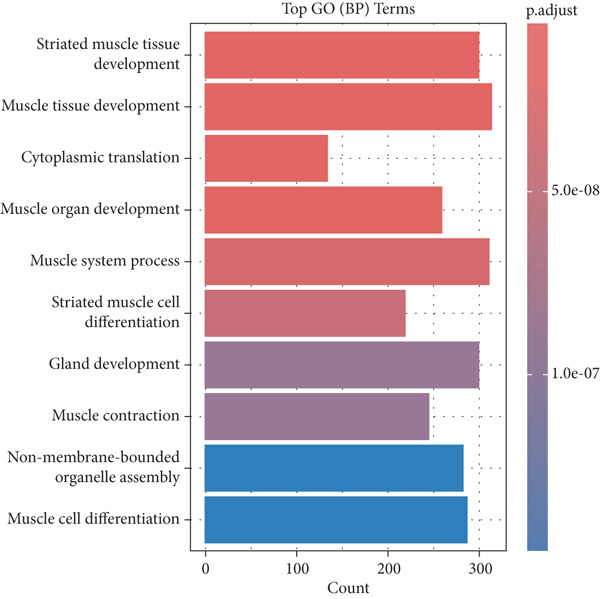
Gene ontology biological process enrichment analysis.

Upregulated DEGs in CT were enriched in collagen fibril organization, ECM remodeling, and immune response regulation attributes providing periodontal structural integrity and repair. The enriched terms in GE reflected an epithelial barrier, a response to pathogen defense, and keratinocyte differentiation, reflecting the protective role of the epithelium (Figure 4).

### 3.8. KEGG Pathway Analysis

**Figure 5:**
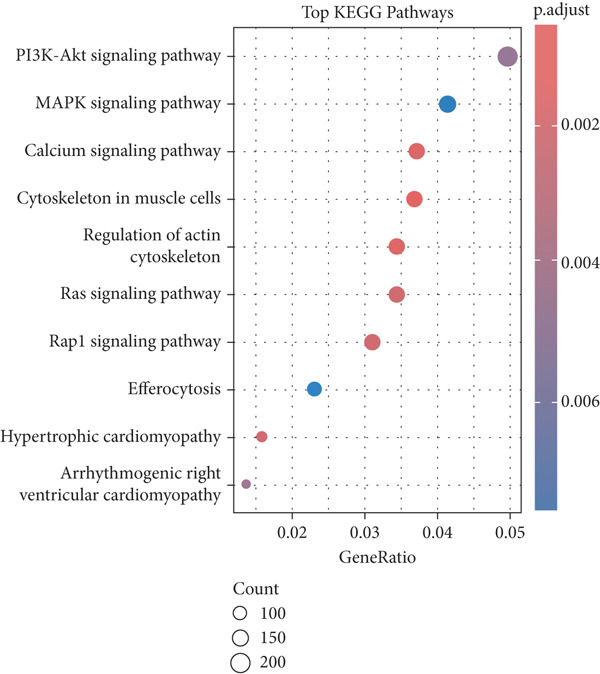
KEGG pathway analysis.

CT‐enriched pathways, such as PI3K–Akt signaling, ECM–receptor interaction, and focal adhesion, are involved in cell–matrix communication and tissue remodeling. GE‐enriched pathways highlighted tight junctions, IL‐17 signaling, and processes that contribute to epithelial homeostasis and immune defense (Figure 5).

### 3.9. Expression of Key Marker Genes

COL1A1 and COL3A1 expression of four representative genes was significantly elevated in CT, which further underscored the importance of collagen biosynthesis and structural support. Consistent with the antimicrobial defense and epithelial differentiation pathways, GE showed elevated levels of DEFB1 and KRT10. These markers were consistent with the broader transcriptional trends indicated by differential expression and enrichment analyses.

### 3.10. Biological Interpretation

In general, these data confirm tissue‐specific transcriptional landscapes where CT seems to have a wider upregulation of ECM maintenance, structural support, and immunomodulation, all central to periodontal homeostasis. GE displays a unique signature of a specialized epithelial barrier, keratinization, AMPs, and tight junction pathways that prevent microbial infiltration. These data underscore the complementary, but not identical, gene expression programs in CT compared with those in GE. CT is transcriptionally biased towards collagen deposition and immune modulation, whereas GE is biased towards barrier integrity and antimicrobial responses.

## 4. Discussion

**Figure 6:**
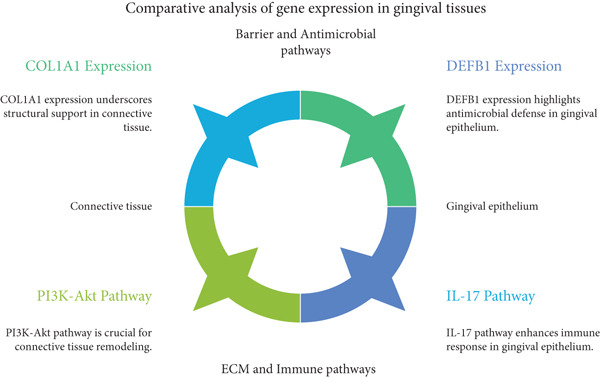
Comparative analysis of gene expression in gingival tissues.

The transcriptomic landscapes for GE and CT are unique, both of which play opposing yet specialized roles in maintaining periodontal health [12]. The molecular insights observed through this analysis of DEGs and enriched pathways corroborate existing literature, while also providing novel insights that extend previous findings. The upregulation in the CT of ECM and collagen‐related genes, such as COL1A1 and COL3A1, is in line with the structural function of CT in periodontal homeostasis. These genes have been implicated as key players in ECM remodeling and the maintenance of CT homeostasis in prior studies [13]. For instance, Bartold and Ivanovski summarized contributions made by ECM proteins to periodontal repair and regeneration, supporting our observations that pathways regarding ECM organization and immune modulation are enriched among CT transcriptomes [14]. The enrichment of KEGG pathways, such as PI3K–Akt signaling and focal adhesion, further highlights the regulation of CT in promoting cell–matrix communication and structural upkeep (Figure 6) [15].

Conversely, GE expressed significantly higher levels of genes involved in epithelial barrier integrity and antimicrobial defense, such as DEFB1 and KRT10. These results confirm those of Dale and Fredericks, who criticized the mark of oral defense, that is, the importance of epithelial AMPs [16]. Moreover, PEA indicated that the transcriptome profile of GE is heavily weighted towards pathways such as tight junctions and IL‐17 signaling, which are necessary for preserving epithelial integrity and instigating immune responses to infection. These findings are consistent with previous studies, which have highlighted the role of tight junction proteins in barrier function in epithelium [17].

A prominent feature of this study was the significant asymmetry between the two tissues in relation to the number of upregulated genes because CT displayed a greater upregulation of the gene repertoire than GE. That difference may be partly because CT is the most versatile and even essential, but least understood tissue in the body, performing functions from sustaining structures to regulating immunity and healing wounds. In contrast, GE is more specialized in antimicrobial defense and preserving barrier integrity [18]. The results of our study also underscore the interrelationship of these tissues in periodontal health, such as the upregulation of immune regulatory pathways in CT, which counterbalances the antimicrobial defense of GE and complements the overall synergy to microbial challenge. This is consistent with previous studies emphasizing the interdependence of periodontal tissues during immunity against pathogenic insults and homeostasis [19].

In fact, the differential expression of immune‐related pathways in both tissues provided insights into their distinct but synergistic roles in immune defense. The enrichment of pathways such as ECM–receptor interaction in CT and IL‐17 signaling in GE suggests that the CT of the gingiva is involved in regulating immune cell trafficking and activation, whereas GE is responsible for the rapid microbial‐prompted response. These findings are in line with the observations made by Hajishengallis and Lambris, who showed how periodontal tissues regulate innate and adaptive immune responses [20]. This study elucidates the unique molecular characteristics of healthy GE and CT and their biological function in maintaining periodontal health. CT, in particular, showed strong upregulation of genes associated with ECM remodeling and immune signaling, indicating its involvement as a mediator in pathways associated with tissue homeostasis, inflammation, collagen biosynthesis, and leukocyte adhesion, as evidenced by the enrichment of these pathways [21].

In contrast, genes related to barrier function and innate immunity were highly expressed in GE. The upregulation of DEFB1, which encodes for *β*‐defensin 1, emphasizes its antimicrobial function in preventing microbial invasion [22]. Other enriched pathways related to keratinocyte differentiation and tight junction signaling highlight the epithelial protective functions in harboring barrier integrity and countering external stress [23]. Such findings are in line with previous investigations, which have proposed tissue‐specific molecular pathways within periodontal tissues. Similar differences in ECM‐associated and immune‐associated pathways in CT and GE have been reported [24]. Nonetheless, this is the first transcriptomic comparison coupled with PEA that expands the knowledge of healthy tissues.

Taken together, the enriched pathways of GE and CT create a synergistic network, which maintains periodontal homeostasis. On the other hand, CT is a structural scaffold that supports tissue integrity and serves as a reservoir for growth factors and immune modulators by upregulating ECM–receptor interactions, PI3K–Akt signaling, and focal adhesion pathways. These routes not only directly affect the biomechanical stability of the gingival CT but also indirectly regulate fibroblast function, collagen turnover, and immune cell recruitment. Concomitant GE in tight junction signaling, IL‐17 signaling, and AMP expression enhances the structural integrity of epithelial barriers to microbial ingress alongside the promotion of rapid immune responses. For the IL‐17 pathway, this would connect epithelial sensing of pathogens with CT‐driven neutrophil and macrophage recruitment and activation bridging surface defense to deeper immune orchestration. This bidirectional communication ensures that barrier breaches are immediately reinforced by the underlying CT, while CT remodeling is kept in check through epithelial‐derived cytokines and chemokines. Moreover, these interactions develop into a functional complex protection from GE acting as the sentinel and CT providing structural and immunological backing, the interaction being crucial to sustain a healthy periodontium.

This study has some limitations but offers new insights. The utilization of publicly available datasets comes with the trade‐off of introducing potential variability between datasets from different lab preparations, sequencing platforms, and data‐processing pipelines. Although batch correction methods have been applied (including ComBat) to reduce such effects, residual technical variability may persist and affect these analyses. Moreover, the results are based on transcriptomic data that require verification by proteomic and functional studies to validate the biological significance of the identified pathways and genes. Despite the valuable insights gained, this study had several limitations. Datasets analyzed were derived from independent studies that could introduce batch differences and differences in sample preparation or sequencing platforms [25]. The results were based exclusively on computational analysis. These findings need to be confirmed by validation on quantitative PCR or immunohistochemistry level [26]. Although this study establishes a database of healthy tissues, it is limited by the lack of demystifying the aforementioned molecular signatures under pathological conditions, such as periodontitis [27]. However, more extensive studies combining multiomics approaches, integrating transcriptomics with proteomics and epigenomics, would provide a more comprehensive picture of the biology of periodontal tissues [28]. Studies investigating gene expression changes over time (longitudinal studies) as periodontal disease progresses could identify key molecular mechanisms that promote tissue destruction. Functional experiments are necessary to confirm the biological importance of the observed DEGs and pathways.

Although the current study provides new insights into transcriptomic signatures of healthy gingival CT and GE, there were some limitations that need to be considered. In particular, the datasets used were generated from various independent studies with different sample collection methods, laboratory preparation protocols, sequencing platforms and computational analysis pipelines. Although batch effect correction was extensive through the ComBat algorithm and supported by a sensitivity analysis using the RUV method that demonstrated very high concordance between methods (Pearson correlation of log_2_ fold‐change values = 0.93), some source of residual technical variability cannot be ruled out entirely. Second, the results were derived entirely from in silico transcriptomic analysis. Experimental validation is needed for the biological significance of the DEGs and enriched pathways. We are currently performing validation studies on the RNA of representative genes (COL1A1, COL3A1, DEFB1, and KRT10) by qPCR in independent gingival tissues, determining their spatial localization by immunohistochemistry (IHC). Integration of transcriptomic studies into proteomic analyses will enhance the translational value of these observations and validate pathway‐level activation. More importantly, this study concentrated exclusively on healthy gingival tissues to establish a normal molecular signature critical for the interpretation of disease‐associated alterations. It is our expectation that future analyses will further enhance this framework by leveraging available transcriptomic datasets from diseases, such as periodontitis and peri‐implantitis, to characterize those molecular changes that are leading to the manifestation and progression of disease. These comparative studies also have great potential for identifying important regulatory pathways and refining therapeutic targets as well as elevating the translational applicability of periodontal transcriptomic profiling.

## 5. Conclusions

This study represents a transcriptomic comparison between healthy GE and CT, highlighting the marked DEG profiles and enriched biological processes. These results contribute to our understanding of the molecular basis of tissue‐specific functions and provide encouragement for future periodontal research. A better understanding of these phenotypes using a combination of computational and experimental approaches may guide the future development of targeted therapies for periodontal disease. Herein, we present a transcriptomic characterization of healthy GE and CT, showing divergent molecular signatures that mediate specialized functions in periodontal health. Coordinated interactions between these tissues, identified by their disparate transcriptomic architectures, are essential for maintaining periodontal homeostasis. These results lay a strong foundation for future studies on the molecular mechanisms affecting periodontal health and diseases, which can identify therapeutic targets that could either prevent or mitigate the destruction of periodontal tissues. Integrating transcriptomic data with proteomic, epigenomic, and metabolomic studies in the future would provide new opportunities to further enhance the understanding of periodontal biology. Experimental validation of the identified genes and pathways will further augment the significance of these observations, setting the stage for the delineation of novel diagnostics and targeted therapeutic approaches for periodontal diseases.

## Consent

Patient consent was not applicable, as there were no human participants involved in this study.

## Conflicts of Interest

The authors declare no conflicts of interest.

## Author Contributions

Conceptualization: Saravanan Sampoornam Pape Reddy and Delfin Lovelina Francis. Methodology: Yash Dhiman and Delfin Lovelina Francis. Investigation: Ankit Gupta and Ruchi Harish. Software and validation: Yash Dhiman and Ankit Gupta. Formal analysis: Manish Rathi and Ruchi Harish. Resources and data curation: Delfin Lovelina Francis and Manish Rathi. Supervision: Shaili Pradhan and Balaji Manohar. Writing – original draft: Saravanan Sampoornam Pape Reddy. Writing – review and editing: Balaji Manohar and Shaili Pradhan.

## Funding

No funding was received for this manuscript.

## Data Availability

Data supporting the findings of this study are available from the corresponding author upon request.
